# The psychometric properties of childhood physical and sexual abuse measures in two Canadian samples of youth and emerging adults

**DOI:** 10.1371/journal.pone.0318448

**Published:** 2025-05-05

**Authors:** Vanessa De Rubeis, Lil Tonmyr, Masako Tanaka, Tracie Afifi, Nicole Catherine, Ana Osorio, Harriet L. MacMillan, Andrea Gonzalez

**Affiliations:** 1 Department of Psychiatry & Behavioural Neurosciences, McMaster University, Hamilton, ON, Canada; 2 Offord Centre for Child Studies, McMaster University, Hamilton, ON, Canada; 3 Centre for Surveillance and Applied Research, Health Promotion and Chronic Disease Prevention, Public Health Agency of Canada, Ottawa, ON, Canada; 4 Department of Community Health Sciences, University of Manitoba, Winnipeg, MB, Canada; 5 Department of Psychiatry, University of Manitoba, Winnipeg, MB, Canada; 6 Children’s Health Policy Centre, Faculty of Health Sciences, Simon Fraser University, Vancouver, BC, Canada; Università degli Studi di Torino: Universita degli Studi di Torino, ITALY

## Abstract

**Introduction:**

Child maltreatment is prevalent in Canada; how we measure it varies. The objective of the current study was to examine the psychometric properties of the Childhood Experiences of Violence Questionnaire Short Form (CEVQ-SF) physical and sexual abuse measures and of the Canadian Community Health Survey (CCHS) 2-item sexual abuse measure, compared with the Childhood Trauma Questionnaire (CTQ) in two samples of adolescents and young adults.

**Methods:**

Retrospective, self-reported child abuse history was collected in the British Columbia Healthy Connections Project (BCHCP) and in the Well-Being and Experiences (WE) Study. Internal consistency, criterion validity, and construct validity were examined.

**Results:**

Across both samples, the prevalence of child physical abuse (CPA) and child sexual abuse (CSA) ranged from 12.5% to 41.4% and from 5.8% to 34.3%, respectively. Internal consistencies were good-to-acceptable for CPA using the CEVQ-SF in the BCHCP (α = 0.83) and the WE Study (α = 0.79) and for CSA using the CEVQ-SF in the WE Study (α = 0.68). For CPA, in both studies, the highest agreement—moderate-to-fair—was between CEVQ-SF severe CPA and CTQ moderate CPA: κ=0.63 (BCHCP) and κ= 0.35 (WE Study). For CSA, agreement with CTQ moderate cut-offs was substantial in the BCHCP (κ=0.77) and fair in the WE Study (κ=0.37).

**Discussion:**

Our findings support current and future use of the CEVQ-SF for CPA, and for CSA, using both the CEVQ-SF and the CCHS-CSA measure, given that they had good psychometric properties when administered to two samples of adolescents and young adults.

## Introduction

Child maltreatment is prevalent in Canada and worldwide [1,2]. It is associated with negative physical and mental health outcomes across the lifespan [3–6] and health-care costs in the billions of dollars annually [7]. An analysis of results of the 2012 Canadian Community Health Survey (CCHS) found that one-third of adults reported having experienced some form of maltreatment in childhood [8]. Development of policies and interventions to prevent child maltreatment requires an understanding of its characteristics, including risk and protective factors, impact, and changes over time [9–11]. Reliable and valid measures are the foundation of robust surveillance and research [12].

The prevalence of child maltreatment varies internationally [13]. In addition to true differences, variations may be related measures selected, modes of administration, and non-methodological issues, such as stigma or fear, or minimization and denial [2,13–15]. A long-standing scarcity of retrospective data in Canada has gradually been rectified with information from national surveys that capture specific types of child maltreatment. However, given the sensitive nature of this data, reports by children and youth are still limited [16].

To estimate the prevalence of child maltreatment, psychometrically sound self-report measures are essential. Recent reviews have highlighted the variety of measures that have been employed, noting the uneven methodological quality of these measures, with only a handful achieving moderate-to-strong ratings [10,11,17–19].

The Childhood Trauma Questionnaire (CTQ) [20] and the Juvenile Violence Questionnaire (JVQ) [21–23] are retrospective, self-reported measures with fairly good psychometric properties. However, broad application of these measures in population-based studies is restricted by their length (administration time ranges from 5 to 30 minutes) and by copyright status, which entails a substantial cost for use (e.g., the CTQ).

The Childhood Experiences of Violence Questionnaire (CEVQ) is a self-report tool that measures child physical abuse, child sexual abuse, and exposure to intimate partner violence that occurred before age 16. It has moderate-to-strong reliability and validity [11,18,24,25]. The CEVQ Short Form (CEVQ-SF), which also measures child physical abuse (CPA) and child sexual abuse (CSA), has good-to-excellent reliability and validity [25].

Previous work has tested the psychometric properties of the CEVQ-SF relative to the CEVQ-long form and the CTQ [25], but little attention has been given to their use with populations such as high-risk subgroups, younger age groups (<15 years), and clinical versus community samples. In addition, Canadian research has been conducted in only one province (Ontario) [26].

Most Statistics Canada surveys including the 2019 General Social Survey, 2018 Survey of Safety and Public and Private Spaces (SSPPS), and the 2022 Mental Health and Access to Care Survey (MHACS), all use two questions to measure child sexual abuse which were initially developed and used in the CCHS-Mental Health 2012, which we refer to at the CCHS-CSA [27] the psychometric properties of which measure have yet to be evaluated. Understanding the psychometric properties of this child sexual abuse measure is of particular interest, as it is included in the 2023 Canadian Healthy Survey on Children and Youth, which is a national survey that will provide a up-to-date picture of the prevalence of maltreatment in Canada [28]. Beyond the Canadian context, the psychometric properties can inform other users if this is an adequate tool for different samples or populations.

The objectives of the current study were to examine the validity and reliability of (1) the CEVQ-SF CPA measure, (2) the CEVQ-SF CSA measure, and (3) the CCHS-CSA measure, when they were administered to a sample of pregnant 14- to 24-year-olds from the British Columbia Healthy Connections Project (BCHCP) and to a sample of youth aged 18–21 years from the Well-Being and Experiences (WE) study in Manitoba. These studies provide an opportunity to compare the psychometric properties of the CEVQ-SF CPA and the CSA measures with those of the CTQ in two diverse Canadian samples.

## Methods

### British Columbia Healthy Connections Project (BCHCP): study design and sample

The BCHCP was a randomized controlled trial (RCT) designed to evaluate the effectiveness of the Nurse-Family Partnership home visitation program in improving maternal-child outcomes in British Columbia, Canada [29,30]. Pregnant 14- to 24-year-olds were eligible to participate if they: (a) were less than 28 weeks gestation; (b) were preparing to parent for the first time; and (c) met criteria for socioeconomic disadvantage. The disadvantage criteria were limited income (receiving income assistance or experiencing homelessness) or affordability challenges with respect to food or rent; limited education (less than high school graduation); and preparing to parent while single. Those aged 14–19 were automatically eligible because of their age; 20- to 24-year-olds had to meet two of the three socioeconomic criteria. Referrals to the BCHCP were made through public health units at four regional BC Health Authorities. A total of 739 girls and young women were enrolled. All participants provided written informed consent after having the consent form read aloud to them to ensure comprehension; mature minors were deemed competent to provide informed consent.

Using standardized protocols [29], baseline data were collected during in-person visits to participants’ homes from October 2013 to December 2016. The baseline data included: sociodemographic characteristics, mental health, history of childhood abuse (before age 16), and exposure to intimate partner violence. Data collection for sensitive topics was addressed by inviting participants to use headphones with audiotaped questions; participants placed their written responses in sealed envelopes for processing by the research team. Participants were randomized to receive either existing services or a nurse home visitation program beginning in pregnancy and lasting until the child was two years old [29]. The RCT study protocol contains additional details [29,30]. Research ethics approvals were obtained from all participating organizations, including Simon Fraser University (2012 s0738), McMaster University (13–570), and the Public Health Agency of Canada (2012–0039).

### Well-Being and Experiences (WE) Study: study design and sample

The WE Study was a longitudinal survey of youth in Manitoba, Canada who were aged 14–17 years (n = 1002) at baseline (Wave 1). The Study used a multi-pronged sampling design that involved random digit dialling of landlines and cellphones (21%) and convenience sampling (79%), which included referrals and community advertisements. Of the random digit dialing sample, 83% of households that were contacted expressed interest in participating; most (97%) were ineligible because no 14- to 17- year-olds lived in the households at the time. Of those eligible, 63% consented and completed the survey. For most variables, including, age, grade, and racial-ethnic identity, no differences were apparent between participants recruited through random digit dialling versus convenience sampling. However, the former were more likely to be in the highest household income category and less likely to report parental separation or divorce. Sex, income, racial-ethnic identity, and postal codes were examined to ensure that the sample represented the population [31].

Data were collected from 2017 to 2022. Baseline Wave 1 data were collected between July 2017 and October 2018. Follow-up waves were conducted at one-year intervals: Wave 2 (2019), Wave 3 (2020), Wave 4 (2021), and Wave 5 (2022).

All waves of data except Wave 2 were used for the current analyses. Child sex and household sociodemographic characteristics were from Wave 1. Youth data came from Waves 3–5 (n = 622), depending on when adolescents reached age 18. For the baseline wave, youth completed a questionnaire in private rooms at a research facility; parents did not have access to youth responses. For follow-up waves, youth received an individualized link to complete the survey online. All participants provided informed consent and were aware that they could withdraw at any time. The Health Research Ethics Board at the University of Manitoba granted ethics approval (HS19968 (H2016:275)).

### Measures

#### History of childhood abuse.

For CPA, both the BCHCP and the WE Study used the CEVQ-SF CPA measure, which consists of three questions about incidents before age 16 [25]. Presence or absence of CPA was dichotomized, based on standardized CEVQ criteria (S1 Table). CPA frequency was assessed on an ordinal scale (never, 1 or 2, times, 3–5 times, 6–10 times, and more than 10 times). For severity, we applied the cut-offs for physical abuse and severe/frequent physical abuse.

For CSA, the BCHCP used one question from the CEVQ-SF; the WE Study used the CCHS-CSA measure (S1 Table). In both studies, questions pertained to incidents that occurred before age 16; response options were: never, 1 or 2, times, 3–5 times, 6–10 times, and more than 10 times. S1 Table contains the standardized cut-offs for the CEVQ-SF and for the CCHS-CSA measure that were applied to dichotomize responses.

Both studies used the Childhood Trauma Questionnaire (CTQ), a 28-item, self-report measure of five types of maltreatment “when growing up [32].” Response options for each type are: never true (score 1), rarely true [2], sometimes true [3], often true [4], and very often true [5]. Items within each type are summed to create a total score for that type, which can be classified into ranges of severity: none, low, moderate, and severe. All items in the CTQ and scoring criteria for each maltreatment type have been published previously [32]. Both the “moderate” and “severe” cut-offs were used where applicable (S2 Table).

#### BCHCP measures of mental distress.

The BCHCP used the Kessler Psychological Distress scale (K10) [33] to measure non-specific psychological distress in the last 30 days [33,34]. The scale consists of 10 items with five Likert-type response categories: none of the time (score 1), a little of the time [2], some of the time [3], most of the time [4], and all the time [5]. Total scores can range from 10 to 50, with higher scores indicating greater distress. In the BCHCP sample, internal consistency was α=0.93.

The RAND Mental Health Inventory (MHI) [35] is a 38-item instrument that assesses levels of depression, anxiety, and well-being. It has two global scales, six subscales, and one total index score. For the current analyses, we used only the Psychological Distress global scale, a sum of 24 items, with higher scores signalling greater distress. In the BCHCP sample, internal consistency was α=0.91.

#### WE Study measures of mental distress.

The WE Study administered two measures of mental distress to youth: the Generalized Anxiety Disorder (GAD-7) [36] and the Patient Health Questionnaire (PHQ-9) [37]. Both require respondents to rate, on a 4-point scale, how bothered they have been by certain problems over the past two weeks. A summed score of 10 or more is considered *clinically significant* and coded as probable depression or anxiety [37,38].

#### Statistical analyses.

Descriptive statistics were used to present sociodemographic characteristics (age, racial-ethnic identity, education, and income), the prevalence of child physical abuse and child sexual abuse types by severity, co-occurrence of child physical abuse and child sexual abuse, and mental distress. Internal consistency, a measure of reliability, for the child abuse measures was assessed using Cronbach’s alpha (α). Criterion validity was assessed by computing Cohen’s kappa (κ) to measure the strength of agreement between each child physical abuse and child sexual abuse measure and the CTQ, based on Landis and Koch [39].

To assess construct validity, the samples were classified into mutually exclusive severity groups. For example, the CTQ child sexual abuse measure was used to create the two severity groups, one using the moderate cutoff and the other using the severe cutoff to determine the presence or absence of child sexual abuse. The BCHCP sample was grouped into [1] no child sexual abuse versus [2] child sexual abuse based on the CEVQ-SF. For the WE Study [1], no child sexual abuse versus [2] child sexual abuse groups were created based on the CCHS-CSA measure; if respondents confirmed either of the two items in the measure, it was identified as child sexual abuse. This process was repeated for child physical abuse (S1 Table). For both studies, t-tests were used to compare severity group differences on continuous measures capturing mental health outcomes. Data analyses were conducted using SAS 9.4 [40] and STATA 17 [41], respectively.

## Results

Table 1 displays the sociodemographic characteristics of the two samples. In the BCHCP sample, participants’ ages ranged from 14 to 24 years (M = 19.8, SD = 2.33). About half had not completed high school (50.5%), including 25% who were still enrolled. Most met the definition of limited income, with a mean annual income of $9,928 (SD = $10,575).

**Table 1:** Sample characteristics from British Columbia Healthy Connections Project (BCHCP) and Well-Being and Experiences (WE) Study.

Variable	N (%) or range, mean (SD)
**BCHCP (n = 682)**	
**Sex**	
Female	682 (100%)
**Age (years)**	14–24, 19.8 (2.33)
**Racial-ethnic identity** ^a^	
Indigenous including First Nations, Métis, and Inuit	68 (10.0)
Indigenous including First Nations, Métis, Inuit, and other	111 (16.3)
Mixed heritage ≥ 2	50 (7.3)
Asian (Chinese, South Asian, Japanese, Filipino, other)	31 (4.6)
Latin-American, Black, other	31 (4.6)
White	391 (57.3)
**Highest level of education**	
None/ess than high school	344 (50.5)
High school or equivalent	262 (38.5)
College diploma or university degree	75 (11.0)
Missing	1
**Income from employment (annual CAD)**	
Less than $5,000	279 (41.7)
$5,000 to less than $9,999	112 (16.7)
$10,000 to less than $19,999	167 (25.0)
$20,000 to less than $29,999	70 (10.5)
$30,000 or more	41 (6.1)
Missing	13
**WE Study (n = 622)** ^b^	
**Sex** ^c^	
Female	344 (55.6)
Male	275 (44.4)
**Age (years)**	18-21, 18.5 (0.76)
**Highest level of education**	
Currently in high school (Grades 10–12)	40 (6.5)
Graduated high school or attained high school equivalency	167 (27.0)
Dropped out of high school	46 (7.4)
Currently in college or university	348 (56.2)
Dropped out of college or university	12 (1.9)
Graduated college or university	6 (1.0)
**Household income^(parental income)^, C** ^a^	
$49,999 or less	199 (19.9)
$50,000 to $99,999	351 (35.1)
$100,000 to $149,999	222 (22.2)
$150,000 or more	180 (18.0)
No response	48 (4.8)

^a^Participants could provide more than one answer.

^b^Because of ethics agreements, we are unable to provide racial-ethnicity demographic information for youth. The protocol stated it would only be used for covariate purposes.

^c^Reported at baseline.

In the WE Study sample, participants’ ages ranged from 18 to 21 years (M = 18.5, SD = 0.76). When the child abuse data were collected (not at baseline), 56% were enrolled in college or university; 27% had completed high school; and 7% were still in high school. Annual household income was based on parental income; at baseline, almost 20% of the sample reported less than $50,000. Descriptives for the mental health measures from both studies are available in S3 Table.

### Prevalence of childhood abuse

Table 2 presents the prevalence of childhood abuse types and the co-occurrence of child physical abuse and child sexual abuse. In the BCHCP sample, the CEVQ-SF captured a higher prevalence of child physical abuse than did the CTQ for the moderate cut-off (41.4% versus 24.2%) and the severe cut-off (30.1% versus 12.5%). Lower child physical abuse prevalence was reported for the WE Study sample, but again, prevalence was higher using the CEVQ-SF rather than the CTQ.

**Table 2:** Prevalence of child physical abuse (CPA), child sexual abuse (CSA), and CPA/CSA co-occurrence, based on CEVQ-SF and CTQ, BCHCP and WE Study samples.

	BCHCP		WE Study	
	CEVQ-SF only N (%)	CTQ only N (%)	CEVQ-SF and CCHS CSA measure only N (%)	CTQ only N (%)
**Total sample**	**682**	**682**	**622**	**622**
**Child abuse type and cutoffs**				
CPA (moderate)	282 (41.4)	165 (24.2)	137 (23.5)	27 (4.4)
CPA (severe/frequent)	205 (30.1)	85 (12.5)	72 (12.5)	6 (1.0)
CSA (moderate)	234 (34.3)	226 (33.1)	35 (5.8)	65 (10.8)
CSA (severe)	–	141 (20.7)	–	25 (4.2)
**Co-occurrence of CPA and CSA (moderate cut-off)**				
No CPA and no CSA	308 (45.2)	386 (56.6)	427 (74.1)	520 (86.4)
CPA only	140 (20.5)	70 (10.3)	116 (20.1)	17 (2.8)
CSA only	92 (13.5)	131 (19.2)	16 (2.8)	56 (9.3)
Both CPA and CSA	142 (20.8)	95 (13.9)	17 (3.0)	9 (1.5)

BCHCP = British Columbia Healthy Connections Project; CPA = child physical abuse; CSA = child sexual abuse; CM = child maltreatment; EA = emotional abuse; EN = emotional neglect; PN = physical neglect; CEVQ-SF = Childhood Experiences of Violence Questionnaire Short Form; CTQ = Childhood Trauma Questionnaire; CCHS = Canadian Community Health Survey; WE study = Well-Being and Experiences study.

In the BCHCP sample, child sexual abuse prevalence was comparable when identified using the CEVQ-SF or the moderate cut-off of the CTQ: 34.3% and 33.1% respectively. In the WE Study sample; child sexual abuse prevalence was higher using the CTQ rather than the CCHS-CSA measure: 10.8% versus 5.8%, respectively.

To compare child physical abuse-child sexual abuse co-occurrence based on the CEVQ-SF versus the CTQ in the BCHCP, we used moderate cut-offs. Owing to the higher prevalence of CPA identified by the CEVQ-SF, co-occurrence was higher based on the CEVQ-SF (20.8%) than on the CTQ (13.9%). For the WE Study sample, the prevalence of co-occurrence was higher when measured by the CEVQ-SF and the CCHS-CSA measure (3.0%) rather the CTQ alone (1.5%).

### Reliability

Internal consistency was good-to-acceptable for child physical abuse using the CEVQ-SF in both the BCHCP and WE Study, α = 0.825 and α = 0.791, respectively. For child physical abuse identified using the CTQ, internal consistency was good in the BCHCP (α = 0.852) and acceptable in the WE Study (α = 0.647).

For child sexual abuse, internal consistency using the CTQ was excellent in both the BCHCP and WE Study: α = 0.943 and α = 0.903, respectively. In the WE study, internal consistency of the CCHS-CSA measure was reasonable (α = 0.676).

### Criterion validity

We examined criterion validity for child physical abuse between the two measures using moderate and severe/frequent cut-offs. The best agreement (moderate-to-fair) was between CEVQ-SF severe/frequent child physical abuse and CTQ moderate child physical abuse: κ=0.631 for the BCHCP, and κ= 0.350 for the WE Study.

For child sexual abuse, agreement between the two measures was very good for the BCHCP: κ=0.770 with CTQ moderate child sexual abuse, and κ=0.615 with CTQ severe child sexual abuse. In the WE Study sample, the kappas indicated fair agreement for the CCHS-CSA measure with both CTQ moderate (κ = 0.365) and severe (κ = 0.320) cut-offs.

### Construct validity

In the BCHCP, moderate and severe/frequent child physical abuse measured using the CEVQ-SF and the CTQ was associated with significantly higher levels of psychological distress (Table 3). In the WE Study, child physical abuse identified using both the CEVQ-SF and the CTQ, regardless of severity, was associated with significantly elevated depressive and anxiety symptoms (Table 3).

**Table 3:** Mental health outcomes, by childhood physical abuse, using CEVQ-SF and CTQ physical abuse cut-offs among the BCHCP and WE Study samples.

CEVQ						
	**Standard cut-off**			**Severe/Frequent cut-off**		
	**No CPA**	**CPA**		**No CPA**	**CPA**	
**BCHCP**	Mean score (SD)	Mean score (SD)	Test statistic	Mean score (SD)	Mean score (SD)	Test statistic
Kessler psychological distress scale (K10)	20.1 (7.6)	23.6 (8.3)	-5.66^***^	20.5 (7.8)	24.0 (8.3)	-5.37^***^
RAND Mental Health Inventory (MHI)	59.4 (20.6)	68.4 (22.0)	-5.29^***^	60.4 (21.0)	69.5 (21.7)	-5.04^***^
**WE Study**						
Depressive symptoms (PHQ-9)	8.1 (5.9)	12.0 (6.7)	-6.37^***^	8.4 (6.0)	13.0 (6.5)	-5.96^***^
Anxiety symptoms (GAD-7)	6.7 (5.5)	9.9 (6.6)	-5.55^***^	7.0 (5.6)	10.4 (7.0)	-4.64^***^
**CTQ**						
	**Moderate cut-off**			**Severe/Frequent cut-off**		
**BCHCP**						
Kessler Psychological Distress Scale (K10)	20.9 (7.9)	23.5 (8.4)	-3.65^***^	21.3 (7.9)	23.4 (8.9)	-2.23^*^
RAND Mental Health Inventory (MHI)	61.3 (21.0)	68.7 (22.7)	-3.80^***^	62.2 (21.0)	69.1 (24.6)	-2.70^**^
**WE Study**						
Depressive symptoms (PHQ-9)	8.8 (6.3)	14.5 (6.4)	-4.45^***^	9.0 (6.3)	18 (6.2)	-3.48^***^
Anxiety symptoms (GAD-7)	7.3 (5.8)	12.2 (6.4)	-4.16^***^	7.4 (5.9)	13.7 (6.2)	-2.56^*^

t-test between No CPA and CPA

*p < .05,

**p < .01,

***p < .001. BCHCP = British Columbia Healthy Connections Project; CPA = child physical abuse; CEVQ-SF = Childhood Experiences of Violence Questionnaire Short Form; CTQ = Childhood Trauma Questionnaire; GAD-7 = Generalized Anxiety Disorder, 7-item; K10 = Kessler Psychological Distress Scale; MHI = RAND Mental Health Inventory; PHQ-9 = Patient Health Questionnaire, 9-item; WE Study = Well-Being and Experiences Study.

BCHCP participants who reported child sexual abuse had significantly high psychological distress, measured using the K10 and the RAND MHI, compared with those reporting no child sexual abuse when measured with the CEVQ-SF (Table 4) and the CTQ (Table 5). Similarly, in the WE Study, child sexual abuse identified using the CCHS-CSA measure and the CTQ was associated with slightly, but significantly, elevated depressive and anxiety symptoms (Tables 4 and 5).

**Table 4:** Mental health outcomes, by childhood sexual abuse, using CEVQ-SF CSA for BCHCP sample and CCHS-CSA measure for WE Study sample.

	No CSA	CSA	
**BCHCP**	Mean score (SD)	Mean score (SD)	Test statistic
Kessler Psychological Distress Scale (K10)	20.7 (7.8)	23.2 (8.4)	-3.98^***^
RAND Mental Health Inventory (MHI)	60.9 (20.9)	67.4 (22.4)	-3.60***
**WE Study**			
Depressive symptoms (PHQ-9)	8.7 (6.1)	14.3 (6.5)	-5.24***
Anxiety symptoms (GAD-7)	7.2 (5.8)	11.3 (5.4)	-4.10***

*Note:* t-test between No CSA and CSA

*p < .05,

**p < .01,

***p < .001. BCHCP = British Columbia Healthy Connections Project; CSA = child sexual abuse; CEVQ-SF = Childhood Experiences of Violence Questionnaire Short Form; CCHS = Canadian Community Health Survey; GAD-7 = Generalized Anxiety Disorder, 7-item; K10 = Kessler Psychological Distress Scale; MHI = RAND Mental Health Inventory; PHQ-9 = Patient Health Questionnaire, 9-item; WE Study = Well-Being and Experiences Study.

**Table 5:** Mental health outcomes, by childhood sexual abuse, using CTQ sexual abuse cut-offs among the BCHCP and WE Study samples.

	Moderate cut-off			Severe cut-off		
**No CSA**	**CSA**		**No CSA**	**CSA**	
**BCHCP**	Mean score (SD)	Mean score (SD)	**Test statistic**	Mean score (SD)	Mean score (SD)	**Test statistic**
Kessler Psychological Distress Scale (K10)	20.8 (7.8)	23.0 (8.4)	-3.36^***^	21.1 (7.9)	23.2 (8.6)	-2.67^**^
RAND Mental Health Inventory (MHI)	61.4 (21.1)	66.6 (22.1)	-2.91^**^	62.2 (21.3)	66.5 (22.7)	-2.02^*^
**WE Study**						
Depressive symptoms (PHQ-9)	8.5 (6.1)	13.2 (6.4)	-5.76^***^	8.9 (6.3)	12.5 (6.3)	-2.74^**^
Anxiety symptoms (GAD-7)	7.0 (5.8)	10.9 (5.8)	-5.07^***^	7.3 (5.9)	11 (6.0)	-3.10^**^

t-test between No CSA and CSA

*p < .05,

**p < .01,

***p < .001. BCHCP = British Columbia Healthy Connections Project; CSA = child sexual abuse; CTQ = Childhood Trauma Questionnaire; GAD-7 = Generalized Anxiety Disorder, 7-item; K10 = Kessler Psychological Distress Scale; MHI = RAND Mental Health Inventory; PHQ-9 = Patient Health Questionnaire, 9-item; WE Study = Well-Being and Experiences Study.

Given the differences in the prevalence of child physical abuse based on the CEVQ-SF versus the CTQ, we examined the characteristics of participants classified by combinations of the two measures. Because the CEVQ-SF child physical abuse severe/frequent cut-off and the CTQ child physical abuse moderate cut-off showed the best agreement in both samples, we based our analyses on these classifications. We created four mutually exclusive groups: (1) no child physical abuse on either measure; (2) child physical abuse only on the CEVQ-SF; (3) child physical abuse only on the CTQ); and (4) child physical abuse on both measures. These groups were analyzed in relation to the mental distress measures. Using ad-hoc t-tests, we compared each child physical abuse group with the No-child physical abuse group (reference), and the three child physical abuse groups with each other (Table 6). In the BCHCP, the CTQ-only group had scores similar to those of the No-child physical abuse group for both mental distress measures. The CEVQ-SF-only and both CEVQ-SF and CTQ groups had similar scores, which were significantly higher for psychological distress, compared with the No-child physical abuse group. In the WE Study depressive, and anxiety symptoms were significantly higher among each child physical abuse group than among the No-child physical abuse group (Table 6).

**Table 6:** Mental health outcomes, by childhood physical abuse, using CEVQ-SF and CTQ to define four mutually exclusive CPA categories among the BCHCP and WE Study samples.

	No CPA^1^ N = 447	CEVQ-SF only N = 70		CTQ only N = 30		Both CEVQ-SF and CTQ N = 135	
**BCHCP**	Mean score (SD)	Mean score (SD)	Test statistic	Mean score (SD)	Test statistic	Mean score (SD)	Test statistic
Kessler Psychological Distress Scale (K10)	20.4 (7.6)	24.3 (7.8)	-3.94^***^	21.9 (7.9)	-1.05	23.9 (8.5)	-4.49^***^
RAND Mental Health Inventory (MHI)	60.1 (20.9)	68.8 (20.1)	-3.15^**^	63.7 (23.3)	-0.89	69.9 (22.5)	-4.57^***^
**WE Study**	**N = 497**	**N = 53**		**N = 6**		**N = 19**	
Depressive symptoms (PHQ-9)	8.30 (6.0)	12.5 (6.5)	-4.64^***^	15.3 (6.5)	-2.30*	14.5 (6.5)	-4.41^***^
Anxiety symptoms (GAD-7)	6.9 (5.6)	9.7 (7.1)	-3.36^***^	12.0 (7.2)	-2.01*	12.2 (6.6)	-4.00^***^

1. Ad-hoc t-test for No CPA on both measures (reference) and each CPA group.

*p < .05,

**p < .01,

***p < .001 BCHCP = British Columbia Healthy Connections Project; CPA = child physical abuse; CEVQ-SF = Childhood Experiences of Violence Questionnaire Short Form; CTQ = Childhood Trauma Questionnaire; GAD-7 = Generalized Anxiety Disorder, 7-item; K10 = Kessler Psychological Distress Scale; MHI = RAND Mental Health Inventory; PHQ-9 = Patient Health Questionnaire, 9-item; WE Study = Well-Being and Experiences Study

## Discussion

This analysis of the psychometric properties of the CEVQ-SF child physical abuse, CEVQ-SF child sexual abuse, and CCHS-CSA measures has important and immediate research and policy implications because these instruments are used to measure history of childhood abuse in Canadian surveys. There is an urgent need for brief, high-quality self-report measures of child maltreatment that can be included in surveys involving children and youth and used consistently over multiple years. The higher prevalence of child physical abuse and child sexual abuse in the BCHCP sample compared with the WE Study sample is not surprising given that the former is a high-risk population. Emergence of the anticipated discrepancy between the two populations reinforce the face validity of the CEVQ-SF for detecting/measuring abuse. Child physical abuse prevalence rates differed depending on whether the CEVQ-SF or the CTQ was used. Variations in the prevalence and low agreement between measures may reflect differences such as questions about specific behaviours rather than generally worded questions and response scales that measure frequency rather than severity [42]. Prevalence differences between the BCHCP and the WE Study may be associated with the items and the response scales [43,44].

The CEVQ-SF is behaviour-based; questions centre on frequency of occurrence: “How many times did … [exposure] happen?” By contrast, the CTQ tends to focus on consequences: “Circle the option that best describes how you feel.” In addition, response options differ. In the CEVQ-SF, the number of times an event occurred ranges from “never” to “10 or more times,” and prevalence cut-offs are based on frequency. In the CTQ, response options range from “never true” to “very often true,” and prevalence cut-offs are based on total subscale scores with standardized cut-offs indicating severity. Differing cut-off criteria can yield varying results [45]. For child physical abuse, both the CEVQ-SF and the CTQ had good-to-acceptable internal consistency in both samples, but the CEVQ-SF child physical abuse severe cut-off had the best agreement with the CTQ moderate cut-off. Prevalence estimates calculated using the CEVQ-SF child physical abuse severe cut-off scores were more closely aligned with rates among population-based samples of the same ages [5,13,46]. The CEVQ-SF child physical abuse severe cut-off was also the most reliable predictor of psychological distress and mental health symptoms in both samples.

For CSA, both the CEVQ-SF and the CCHS-CSA measures showed reasonably robust psychometric properties. The moderate-to-strong psychometric properties of the CCHS-CSA measure are noteworthy because it has not been tested against other measures of child sexual abuse.

Different tools may capture different patterns and types of maltreatment, and thereby, contribute to the development of targeted prevention and intervention strategies [10]. For instance, the psychometric properties of the CEVQ-SF child physical abuse were tested in samples with substantially different characteristics, which suggests that the measure is suitable for different study populations. It is important to test different measures of child maltreatment among different samples as this can help to inform the accuracy and applicability to different subgroups [47]. Continued research, including qualitative studies, are needed to explore why differences in maltreatment rates exist among different samples, which will contribute to a better understanding of the applicability of different child maltreatment measurement tools.

When designing child maltreatment research, considerations of administration costs, time, and respondent burden affect the choice of measurement instruments. This analysis shows the CEVQ-SF to be a robust measure of the prevalence of child physical abuse and child sexual abuse. As well, the CEVQ-SF is short and easy to administer, and because it is not copyrighted, does not entail additional costs, meaning it is a feasible and accessible tool [16]. Understanding the psychometric properties of this tool is important to ensure accurate and valid measurement of child maltreatment among different subgroups across different settings [48].

### Strengths and limitations

The examination of the reliability and validity of the CEVQ-SF across two distinctly different samples of young Canadians from two provinces is a strength of this analysis. However, it is important to interpret comparisons with caution given the differences in samples (e.g., only females included in BCHCP), however, results from each study can be generalized to samples who share similar characteristics to those included in the studies. Using two samples also allowed for these measures to be tested among different groups of youth and emerging adults, offering a unique opportunity. Using relatively few questions to assess child physical abuse and child sexual abuse reduces the response burden and the time needed for data collection. Another strength is that both study samples included respondents as young as 14, who are not typically asked to recall experiences of maltreatment. Our findings suggest that the measures we investigated are appropriate for administration to adolescents. No participants in either study reported distress or requested support services due to unsettling questions to the research staff. These findings are consistent with the literature [16]. For child sexual abuse, the WE Study collected data using the CCHS-CSA measure, which was originally designed for adults. The WE Study presented an opportunity to test the psychometric properties of this measure when applied to a young population. A major shortcoming is that we were unable to examine the psychometric properties of other forms of child maltreatment (emotional abuse, neglect, exposure to intimate partner violence) using the CEVQ-SF and the CCHS CSA, and given the limited age range, and the BCHCP sample only included females, we were unable to further explore sex and age differences. Current and future studies using Statistics Canada data (e.g., GSS, SSPPS or 2022 MHACS) should stratify by sex and age groups to examine prevalence rates and impact on outcomes over various ages and cohorts. Additionally, because the samples consisted only of selected residents of British Columbia and Manitoba, generalizability is limited. However, these measures were previously tested in Ontario, and the current study adds to the evidence base [5,25].

## Conclusion

Our analysis supports the use of the CEVQ-SF to estimate CPA prevalence (based on the severe-cut off) and both the CEVQ-SF CSA and the CCHS-CSA to estimate the prevalence of CSA. Compared with the CTQ, these two instruments have good reliability and validity and are shorter, cost-effective measures of child maltreatment. As well, research is underway to establish minimum frequencies to define each type of child maltreatment and determine correlates with health outcomes. Our results provide evidence for statistically informed cut-points. Increasingly, these measures are being included in national surveys; an understanding of their psychometric properties can contribute to child maltreatment research in Canada.

## Supporting information

S1 Table Child physical and sexual abuse: measures, items, and cut-offs. (DOCX)

S2 Table Childhood Trauma Questionnaire (CTQ): Child physical and sexual abuse cut-offs. (DOCX)

S3 Table Mental health measures in BCHCP and WE Study samples. (DOCX)
