# Supplementary material for: The psychometric properties of childhood physical and sexual abuse measures in two Canadian samples of youth and emerging adults
Source: PLoS One. 2025 May 5;20(5):e0318448. doi: 10.1371/journal.pone.0318448 (PMC12052104; doi:10.1371/journal.pone.0318448)
Supplement: S1 Table — (DOCX) [file pone.0318448.s001.docx]

**SUPPLEMENTAL TABLES**

**S1 Table***.* Child physical and sexual abuse: measures, items, and cut-offs

| **Measures** | **Subtype** | **Items: Before age 16, …** | **Moderate/Standard cut-off** | **Severe/Frequent cut-off** |
| --- | --- | --- | --- | --- |
| **CEVQ** | **Physical abuse (BCHCP, WE Study)** | did an adult slap you on the face, head or ears or hit or spank you with something like a belt, wooden spoon, or something hard?  push, grab, shove or throw something at you to hurt you?  kick, bite, punch, choke, burn you, or physically attack you in some way? | 3-5 times or more  3-5 times or more  1-2 times or more | More than 10 times  More than 10 times  1-2 times or more |
| **CEVQ** | **Sexual abuse (BCHCP)** | how many times did an adult touch you in a sexual way when you didn't want them to: for example, touch the private parts of your body or make you touch their private parts, threaten, or try to have sex with you or sexually force themselves on you? | 1-2 times or more | --- |
| **CCHS CSA measure** | **Sexual abuse (WE Study)** | how many times did an adult force you or attempt to force you into any unwanted sexual activity, by threatening you, holding you down or hurting you in some way?  how many times did an adult touch you against your will in any sexual way? By this, I mean anything from unwanted touching or grabbing, to kissing or fondling. | 1-2 times or more  1-2 times or more | --- |

CEVQ=Childhood Experiences of Violence Questionnaire; CCHS=Canadian Community Health Survey; WE Study =Well-Being and Experiences Study
