# Supplementary material for: The psychometric properties of childhood physical and sexual abuse measures in two Canadian samples of youth and emerging adults
Source: PLoS One. 2025 May 5;20(5):e0318448. doi: 10.1371/journal.pone.0318448 (PMC12052104; doi:10.1371/journal.pone.0318448)
Supplement: S2 Table — (DOCX) [file pone.0318448.s002.docx]

**S2 Table.** Childhood Trauma Questionnaire (CTQ): Child physical and sexual abuse cut-offs

|  | **Moderate/Severe cut-off** | **Severe/Extreme cut-off** |
| --- | --- | --- |
| **Child physical abuse** | 10-12 | >=13 |
| **Child sexual abuse** | 8-12 | >=13 |

Bernstein, D. P., Fink, L., Handelsman, L., Foote, J., Lovejoy, M., Wenzel, K., Sapareto, E., & Ruggiero, J. (1994). Initial reliability and validity of a new retrospective measure of child abuse and neglect. *The American Journal of Psychiatry*, 151(8), 1132–1136. https://doi.org/10.1176/ajp.151.8.1132
