# Supplementary material for: The psychometric properties of childhood physical and sexual abuse measures in two Canadian samples of youth and emerging adults
Source: PLoS One. 2025 May 5;20(5):e0318448. doi: 10.1371/journal.pone.0318448 (PMC12052104; doi:10.1371/journal.pone.0318448)
Supplement: S3 Table — (DOCX) [file pone.0318448.s003.docx]

**S3 Table.** Mental health measures in BCHCP and WE Study samples

|  | **Overall sample** |
| --- | --- |
| **BCHCP (N=682)** | **M (SD)** |
| Kessler Psychological Distress Scale (K10, range 10-50) | 21.5 (8.1) |
| RAND Mental Health Inventory (MHI, range 25-142) | 63.1 (21.6) |
| **WE Study (N=622)** |  |
| Depressive symptoms (PHQ-9, range 0-27) | 9.1 (6.4) |
| Anxiety symptoms (GAD-7, range 0-21) | 7.5 (5.9) |

BCHCP=British Columbia Healthy Connections Project; GAD-7=Generalized Anxiety Disorder, 7-item; K10=Kessler Psychological Distress Scale; MHI=RAND Mental Health Inventory; PHQ-9=Patient Health, 9-item Questionnaire; WE Study= Well-Being and Experiences Study
